# Long-term follow-up after en bloc resection of the distal radius with reconstruction using ulnar translocation

**DOI:** 10.1080/23320885.2025.2590296

**Published:** 2025-11-27

**Authors:** Yoshiaki Ogawa, Hisaki Aiba, Yohei Kawaguchi, Satoshi Yamada, Hiroaki Kimura, Yusuke Hattori, Makoto Yamaguchi, Hideki Murakami, Hideki Okamoto

**Affiliations:** ^a^ Department of Orthopaedic Surgery, Nagoya City University Graduate School of Medical Sciences, Nagoya, Aichi, Japan; ^b^ Department of Orthopaedic Surgery, Chita Kosei Hospital, Chita-gun, Aichi, Japan; ^c^ Department of Rehabilitation Medicine, Nagoya City University Graduate School of Medical Sciences, Nagoya, Aichi, Japan

**Keywords:** Distal radius, giant cell tumor, ulnar translocation

## Abstract

Reconstruction of the distal radius after en bloc bone tumor resection is challenging. Among various surgical reconstruction methods, ulnar translocation is a simple approach that does not require vascular anastomosis, autograft harvesting, or prosthesis preparation. This report describes the 7-year follow-up of a patient who underwent reconstruction with ulnar translocation following resection of a recurrent giant cell tumor of the bone. A 57-year-old woman was diagnosed with multiple recurrent giant cell tumor of the bone involving the distal radius. The patient underwent en bloc resection of the tumor with osteotomy of the distal radius 5 cm proximal to the wrist joint through a dorsal incision. The distal ulna was osteotomized at the same level and translocated with preservation of the vascularity of the posterior interosseous artery. Then, the translocated ulna was fixed to the carpal bone and distal radius and aligned in the mid-supination and pronation positions; fixation at 10° of wrist dorsiflexion was performed using locking plates. Bone union between the metacarpal bone, grafted bone and proximal radius was achieved at 9 months postoperatively. At the 1-year follow-up examination, the range of motion of the wrist was 90°/65° (supination/pronation), and the grip strength was 9.1 kg. At the final follow-up examination (7 years postoperatively), the range of motion of the wrist was 90°/90° (supination/pronation) and the grip strength was 19 kg (20 kg on the lateral side). The patient’s QuickDASH and Hand 20 scores were 25 and 43, respectively, indicating minor difficulties in daily activities. Ulnar translocation is regarded as a practical alternative to more complex reconstructive procedures for the distal radius following en bloc tumor resection. Its benefits include surgical simplicity, long-term durability, and preservation of forearm rotation.

## Introduction

Reconstruction of the distal radius following en bloc bone tumor resection is challenging [1]. Various surgical reconstruction methods have been proposed; however, no optimal surgical technique has been established [2]. Wrist arthrodesis has been used to achieve wrist joint stability, grip strength and long-term durability following reconstruction [1]. This technique can be modified for ulnar translocation [3], massive radius osteoarticular allograft arthrodesis [4], the iliac crest [5] or fibular autograft reconstruction [6]. In contrast, wrist arthroplasty is an alternative technique that may improve wrist joint movement [1]. Using this technique, bone defects can be reconstructed using osteoarticular allografts [7], wrist prostheses [8,9] and fibular autografts or allografts [10–12].

A systematic review compared the outcomes of 343 wrist arthrodesis procedures and 618 wrist arthroplasty procedures for rheumatoid arthritis reported by 23 studies that included follow-up for a minimum of 12 months and found that the average increase in grip strength was 76% with arthrodesis (177 patients) and 31% with arthroplasty (330 patients) [13]. Wrist arthrodesis resulted in a loss of range of motion (ROM) across flexion/extension, but pronation/supination motion was maintained at an arc of 145° [13]. In contrast, the ROM of flexion/extension was improved to 60°, and pronation/supination was improved to an arc of 160° [13]. The overall complication rate of arthrodesis was 17% (59 complications in 343 procedures), and complications included carpal tunnel syndrome, prosthetic loosening, tendon adhesions, extensor tendon irritation, wound dehiscence and infections; however, the complication rate of arthroplasty was 19% (111 complications in 600 procedures) and complications included implant dislocation, loosening, wound infection, carpal tunnel syndrome, stiffness and impingement [13]. Because no significant differences between surgical procedures other than ROM restriction were observed, the reconstruction method should be selected based on its long-term durability as well as the patient’s lifestyle, background and preferences.

This report describes the long-term follow-up of a patient who underwent reconstruction with ulnar translocation following a recurrent giant cell tumor of the bone (GCTB) comprising the distal radius that resulted in favorable outcomes.

## Patient

**Figure 1.**
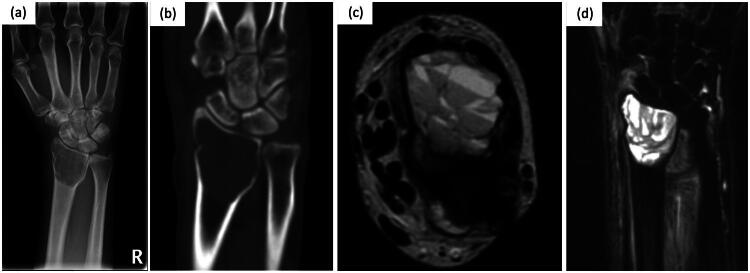
Radiograph of the anteroposterior view (a) at presentation. Coronal computed tomography (CT) image shows cortical bone thinning and microfractures (b). Axial T2-weighted image (c) and T2 fat-saturated sequence (d) show a cystic lesion and fluid-fluid level.

A 57-year-old right-hand-dominant woman presented with a 1-year history of unexplained right wrist pain, which acutely worsened after swinging a golf club. Radiography revealed a pathologic fracture with a lytic lesion on the distal radius without malignant features (Figure 1(a–d)). The patient underwent cyst curettage; however, the surgical specimen indicated no definite diagnosis. Curettage was repeated at 1.5 and 3 years after the initial surgery because of the tumor and pain, and GCTB was confirmed as the diagnosis. The patient experienced a distal radius fracture and pain associated with a third tumor recurrence at 6 years postoperatively. Therefore, en bloc resection with wrist joint reconstruction was planned. A timeline of multiple recurrences is depicted in the Supplemental Figure.

**Figure 2.**
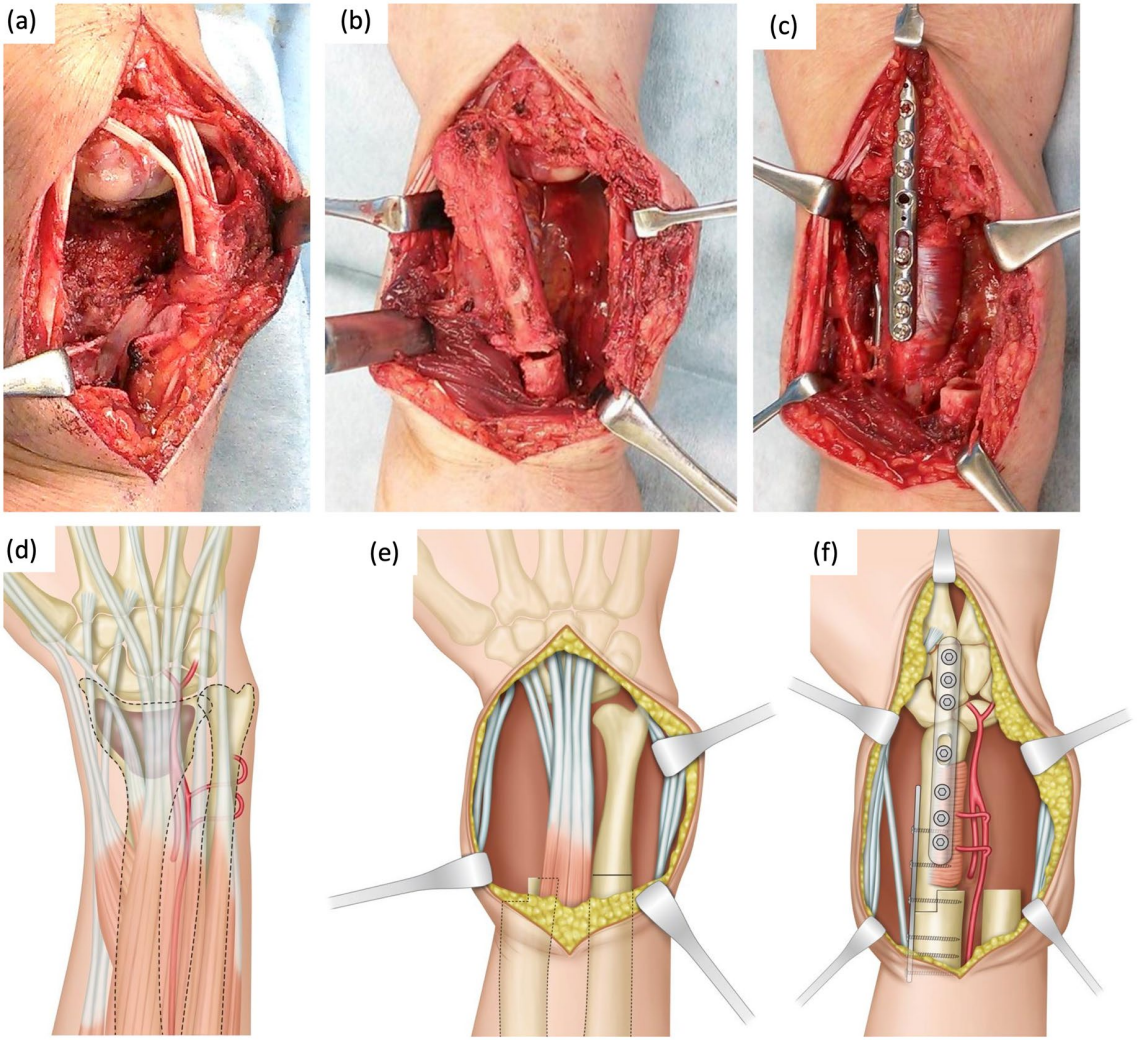
Intraoperative images and corresponding schemas. (a) En bloc resection. (b) Osteotomy of the adjacent ulna. (c) Fixation of the translocated ulna. (d) Schema of the tumor (brown). (e) Schema after osteotomies of the distal radius and ulna. (f) Schema of fixation of the translocated ulna.

During surgery, a dorsal incision was made and the distal radius was exposed by releasing the tendons of the extensor digitorum and extensor pollicis longus [3]. En bloc resection of the distal radius was performed 5 cm from the wrist joint. The adjacent ulna was exposed while preserving the posterior interosseous artery and osteotomized to match the level of the distal radius. The ulna was translocated and the pronator quadratus was retained. The cancellous surface of the styloid process was exposed and trimmed to fit the carpal bone. The scaphoid and lunate cartilage were denuded to create the mortise for insertion of the distal ulna using a diamond bar. After a step-shaped cut of the distal radius and the distal part of the transposed ulna was performed to stabilize the bones, the ulna was aligned in the mid-supination and pronation positions and fixed at 10° of wrist dorsiflexion with locking plates from the proximal third metacarpal bone to the proximal radius *via* the translocated ulna (Figure 2(a–f)).

The forearm was placed in a plaster cast for 1-week postoperatively. Hand therapists trained the patient to perform passive and partially assisted finger and elbow motions. A wrist-supportive orthosis was used for 6 months postoperatively.

In accordance with our institution’s policy and the guidelines of our Institutional Review Board, ethical approval was not required to publish this case report. The procedures used for this case adhered to the principles of the Declaration of Helsinki.

## Results

**Figure 3.**
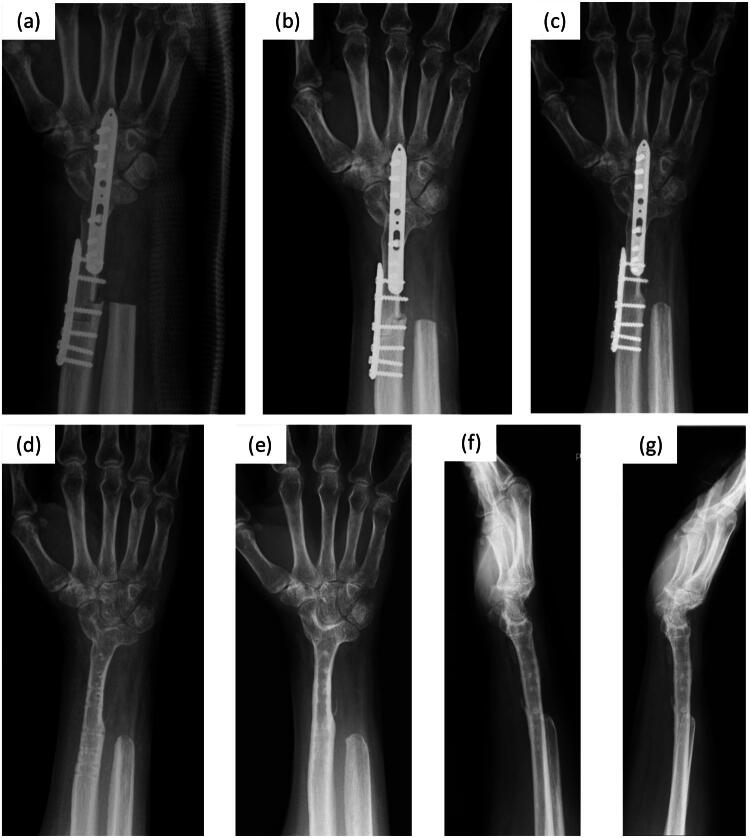
Postoperative radiographs. (a) After surgery. (b) Three months postoperatively. (c) Bone union is observed 9 months postoperatively. (d) After implant removal (9 months postoperatively). (e–g) Anteroposterior view (e), palmar flexion (f), and dorsiflexion (g) at 7 years postoperatively. Subtle wrist motions at the distal carpal alignment are observed.

**Figure 4.**
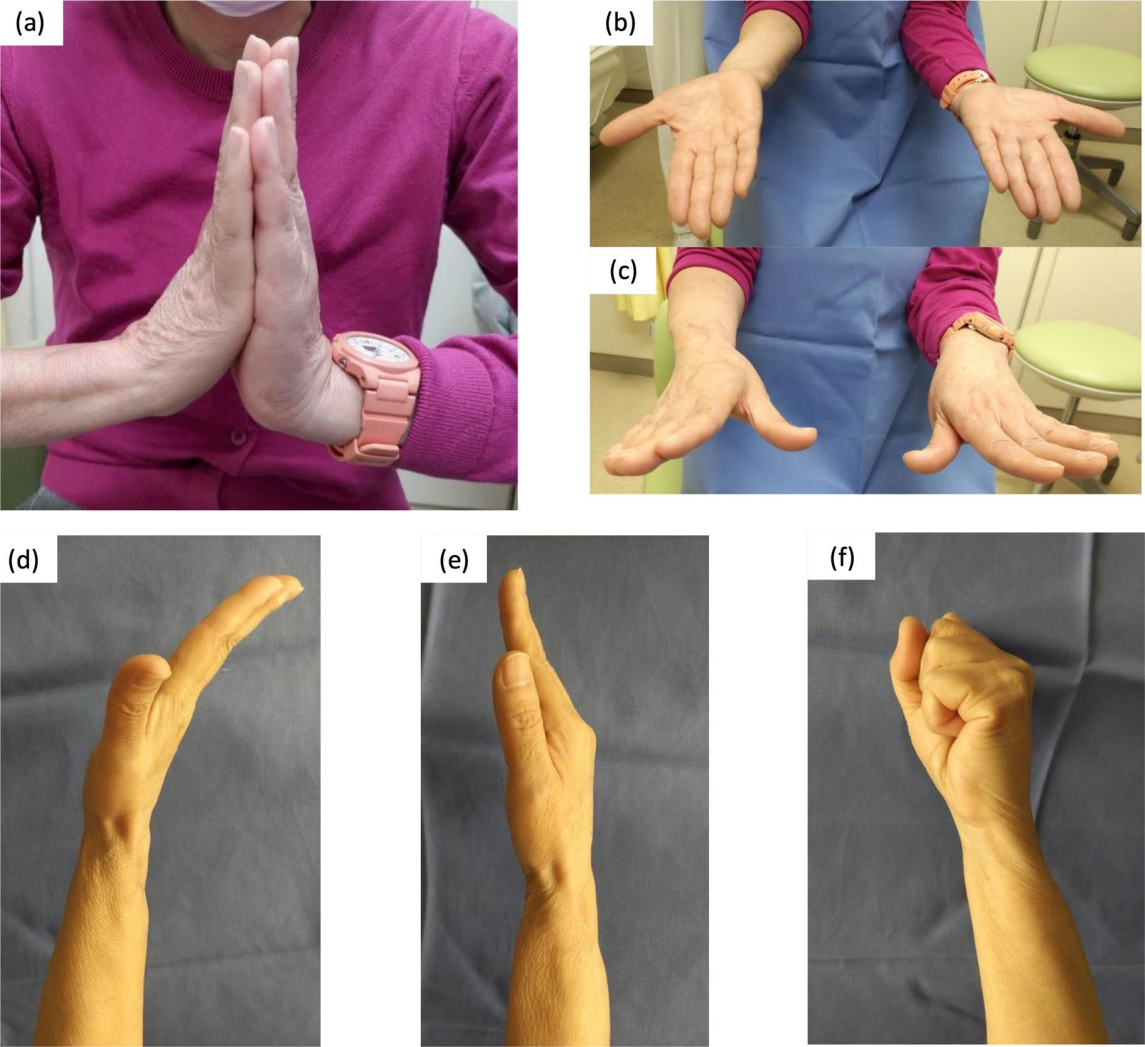
Postoperative function at 7 years postoperatively. (a) Hands in prayer position. (b) Supination (90°). (c) Pronation (75°). (d, e) Dorsiflexion and palmar flexion are possible through movement of the midcarpal and carpometacarpal joints. (f) Grip motion.

The ROM of the wrist during pronation and supination gradually improved postoperatively (Table 1). The bone union between the metacarpal bone, grafted bone and proximal radius was observed at 9 months after reconstruction (Figure 3(a–g)), prompting plate removal (which was strongly demanded by the patient). Local recurrence was noted around the dorsal soft tissue at 5 years after reconstruction (11 years after the first surgery); therefore, the recurrent tumor was removed and the graft and surrounding muscles were preserved. At 7 years after reconstruction (13 years after the first surgery), the QuickDASH [14] and Hand 20 [15] scores were 25 and 43, respectively. The patient resumed a normal life and continued her job as a nurse; however, she experienced some difficulty opening tight jars and mild challenges with household chores (Figure 4(a–f)).

**Table 1.** Postoperative function recovery.

	Time from en bloc resection with ulnar translocation							
	0 weeks	2 months	6 months	1 year*	3 years	4.7 years	5.7 years**	7.3 years
Range of motion (supination/pronation, degrees)	35/35	85/45	90/70	90/65	95/75	95/75	95/75	90/90
Grip strength (kg)***	0	0	9.1 (20.7)	11.9 (21.7)	13.6 (21.7)	NE	17.3 (20.6)	19.0 (20.0)

*The plate was removed 10 months after resection.

**Because of local recurrence, the recurrent tumor was resected 5.9 years after reconstruction.

***Grip strengths on the lateral side are shown in parentheses.

NE: not examined.

## Discussion

GCTB is a locally aggressive and rarely metastasizing tumor comprising mononuclear stromal cells and osteoclast-like giant cells [16,17]. The distal radius is the third most affected site, accounting for approximately 10% of all cases [16,17]. The distal radius is considered a challenging site because of the complex anatomy of the adjacent tendons, nerves, and arteries [18]. Tumor curettage with bone grafting is commonly attempted for patients with Campanacci grade 1 or 2 GCTB to preserve the wrist joint; however, a high risk of local recurrence (30%–50%) has been reported [16–20]. For more advanced cases (Campanacci grades 2 and 3) or multiple recurrent GCTB (such as the current case), en bloc resection of the distal radius is considered [16,17,19–21]. A meta-analysis of six relevant articles that included 80 and 59 patients who underwent curettage and en bloc resection, respectively, reported that patients in the curettage group (relative risk = 2.80), especially those with Campanacci grade 3 (relative risk = 4.90), had higher recurrence rates than those of patients in the en bloc resection group [19].

Wrist joint reconstruction is an important issue after en bloc resection. Ulna translocation was first reported by Groves et al. for treating distal radius nonunion with infection [22]. Subsequently, Seradge et al. reported the first application of this procedure for GCTB in 1982 [23]. By preserving either the anterior or posterior interosseous artery as a nutrient vessel for the distal ulna, the ulna can be used as a pedicle bone graft [24]. Ulnar translocation offers several advantages over other reconstruction methods, including preservation of the donor site and a high rate of bone union because of preservation of the blood supply; additionally, ulnar translocation is more concise than other methods that require vascular anastomosis [24]. However, its disadvantages include the fragility of the wrist supported by a single ulna, instability of the distal ulnar resection stump, and cosmetic issues caused by the thin distal forearm [25]. However, our patient did not express any cosmetic concerns, and no problems related to the fragility of the wrist caused by the single-bone structure were observed. A retrospective study of 25 patients with GCTB treated with ulnar translocation and wrist arthrodesis showed good range of pronation and supination with Musculoskeletal Tumor Society scores of 80% [25]. During a mean follow-up of 23 months, complications included one surgical site infection, one union failure, and two ulnar graft fractures [25]; however, the long-term durability of the reconstruction has not been fully elucidated. During the 7-year follow-up period, the translocated ulna of the current case did not exhibit any atrophy, absorbance, or adjacent bone degeneration.

As an alternative method, wrist arthroplasty offers better wrist motion [7–12], and fibular autografts and allografts are widely used for reconstruction. Saini et al. reported 12 patients with distal radius GCTB who underwent wide resection and wrist arthroplasty using the fibular head [2]. Compared with the contralateral normal side, the affected side retained an average total ROM of 64% (29%–78%) [2]. However, three patients developed wrist subluxation, resulting in pain and functional impairment; these cases were managed with removable wrist splints worn at night and as needed during the day to relieve pain [2]. Because of the possibility of osteoarthritic changes and joint destruction following wrist arthroplasty, patients should avoid heavy labor and minimize excessive loads on the wrist [26]. Regarding the current case, because our patient worked as a nurse, which involves moderately intense hand use, we opted for a joint stabilization procedure rather than arthroplasty to reduce the potential risk of long-term joint laxity.

## Conclusion

Our patient underwent distal radius reconstruction with ulnar translocation for multiple recurrent GCTB. This procedure preserved the function of the forearm and hand during pronation and supination, resulting in satisfactory functional outcomes. Because of its simplicity and long-term durability, this surgical technique could be considered for wrist reconstruction after distal radius resection. However, this surgical technique should be carefully selected after considering the patient’s background and the institution’s experience.

## Supplementary Material

supple.tiff

## Data Availability

Not applicable.
