# Supplementary figures and images for: Long-term follow-up after en bloc resection of the distal radius with reconstruction using ulnar translocation
Source: Case Reports Plast Surg Hand Surg. 2025 Nov 27;12(1):2590296. doi: 10.1080/23320885.2025.2590296 (PMC12667343; doi:10.1080/23320885.2025.2590296)

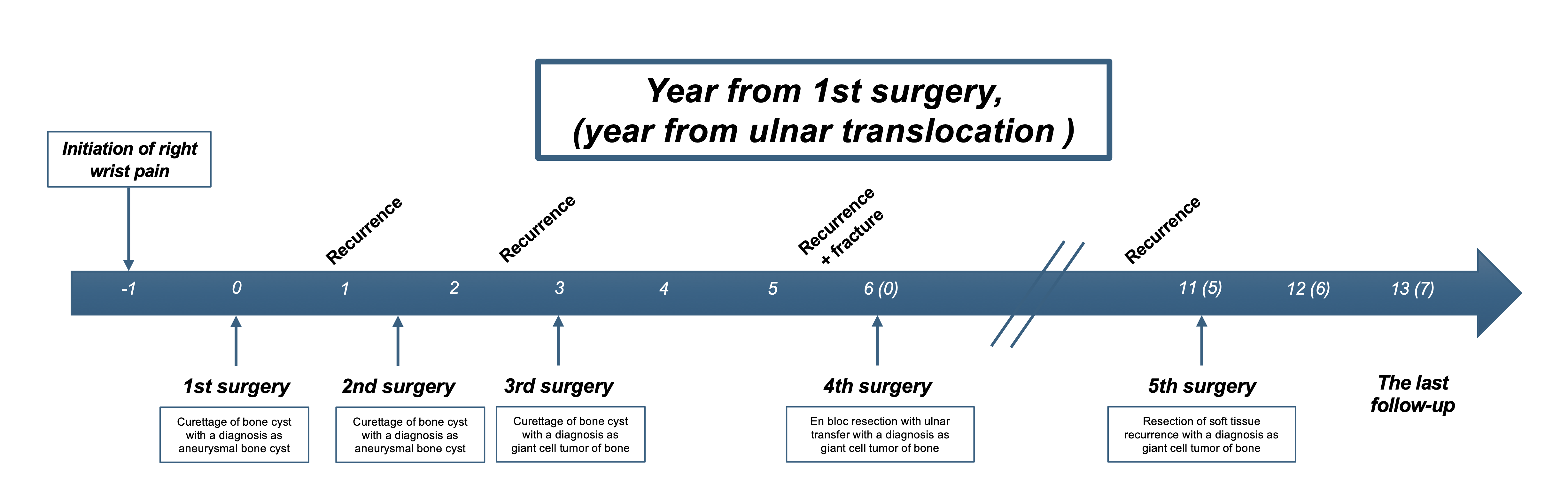

Supplement: supple.tiff [file ICRP_A_2590296_SM8492.tiff]
